# 770. HIV Pre-Exposure Prophylaxis (PrEP) and Post-Exposure Prophylaxis (PEP) knowledge among Medical Students in the Dominican Republic: Are we missing the role of Future Healthcare Provider in PrEP and PEP roll out?

**DOI:** 10.1093/ofid/ofad500.831

**Published:** 2023-11-27

**Authors:** Hector J Lora, Bismarck S Bisono Garcia, Leandro Tapia, Robert Paulino-Ramírez

**Affiliations:** Instituto de Medicina Tropical & Salud Global, Universidad Iberoamericana, UNIBE, Santo Domingo, Distrito Nacional, Dominican Republic; Mayo Clinic, Rochester, Minnesota; Instituto de Medicina Tropical & Salud Global, Universidad Iberoamericana, Santo Domingo, Dominican Republic, Santo Domingo, Distrito Nacional, Dominican Republic; Universidad Iberoamericana, Santo Domingo, Distrito Nacional, Dominican Republic

## Abstract

**Background:**

HIV prevention continues to be a care gap in developing countries, like Dominican Republic. The lack of sufficient programs for prescription and information of general populations are barriers for better control of HIV infection in this country. The knowledge of HIV prevention strategies such as Pre-Exposure Prophylaxis (PrEP) or Post-Exposure Prophylaxis (PEP) among medical professionals and medical students is unknown. The goal of ending HIV Pandemic by 2030 is to rely on prevention strategies.

Our study aims to determine the level of knowledge of medical students in a pre-preclinical cycle and in a clinical cycle regarding HIV/AIDS, PrEP and PEP

**Methods:**

We survey medical students in the Dominican Republic to measure knowledge on HIV, PrEP/PEP. We compared mean knowledge for each topic based on a score out of 10. We analyzed mean scores by using T-Tests and ANOVA with Post Hoc or Tukey Analysis, assuming significance when p< 0.05.

**Results:**

A total of 503 students participated in the survey. ANOVA results showed that there was a statistically significant difference in mean knowledge scores for HIV, PEP, and PrEP (F (2, 1506) = [196.6], p < 0.001). Post hoc analysis revealed that medical students had a higher mean score for HIV knowledge (M= 8.9) than mean scores for PrEP (M= 7.6, p< 0.01) and PEP (M= 7.1, p< 0.01). Similarly, the difference in knowledge scores for PrEP was significantly higher than for PEP (p< 0.01). We further divided participants into Preclinical Years and Clinical years and found that participants within the Clinical Years of Med School had a significantly increased mean PEP knowledge (m= 7.5) than Preclinical Years participants (m= 6.7) (t (460) = 5.8, p< 0.001). Similar results were not appreciated with PrEP, where Clinical year participants had a non-significant increased mean knowledge (m=7.8) compared to their counterparts (m= 7.5) (t (486) = 1.4, p= 0.79).

**Conclusion:**

Our findings might suggest that medical students acquire more knowledge about PEP as they progress into clinical training, but that PrEP knowledge may be emphasized equally in both pre-clinical and clinical training. Further studies should focus on identifying points for improvement and implementing strategies to tackle barriers. One of several limitations of this study is a response bias.

**Disclosures:**

**All Authors**: No reported disclosures

